# Osborne’s Ligament: A Review of its History, Anatomy, and Surgical Importance

**DOI:** 10.7759/cureus.1080

**Published:** 2017-03-06

**Authors:** Andre Granger, Juan P Sardi, Joe Iwanaga, Thomas J Wilson, Lynda Yang, Marios Loukas, Rod J Oskouian, R. Shane Tubbs

**Affiliations:** 1 Department of Anatomical Sciences, St. George's University School of Medicine, Grenada, West Indies; 2 Neurociencias, Pontificia Universidad Javeriana; 3 Seattle Science Foundation; 4 Department of Neurosurgery, University of Michigan, Ann Arbor, Michigan, USA; 5 Neurosurgery, Complex Spine, Swedish Neuroscience Institute; 6 Neurosurgery, Seattle Science Foundation

**Keywords:** pain, peripheral neuropathy, entrapment neuropathy, surgical anatomy, history, ulnar nerve, osborne's ligament

## Abstract

When discussing the pathophysiology of ulnar neuropathy, Geoffrey Vaughan Osborne described a fibrous band that can be responsible for the symptoms seen in this disorder. In this paper, we take a glimpse at the life of Osborne and review the anatomy and surgical significance of Osborne’s ligament. This band of tissue connects the two heads of the flexor carpi ulnaris and thus forms the roof of the cubital tunnel. To our knowledge, no prior publication has reviewed the history of this ligament, and very few authors have studied its anatomy in any detail. Therefore, the aim of the present paper is to elucidate this structure that is often implicated and surgically transected to decompress the ulnar nerve at the elbow.

## Introduction and background

**Figure 1:**
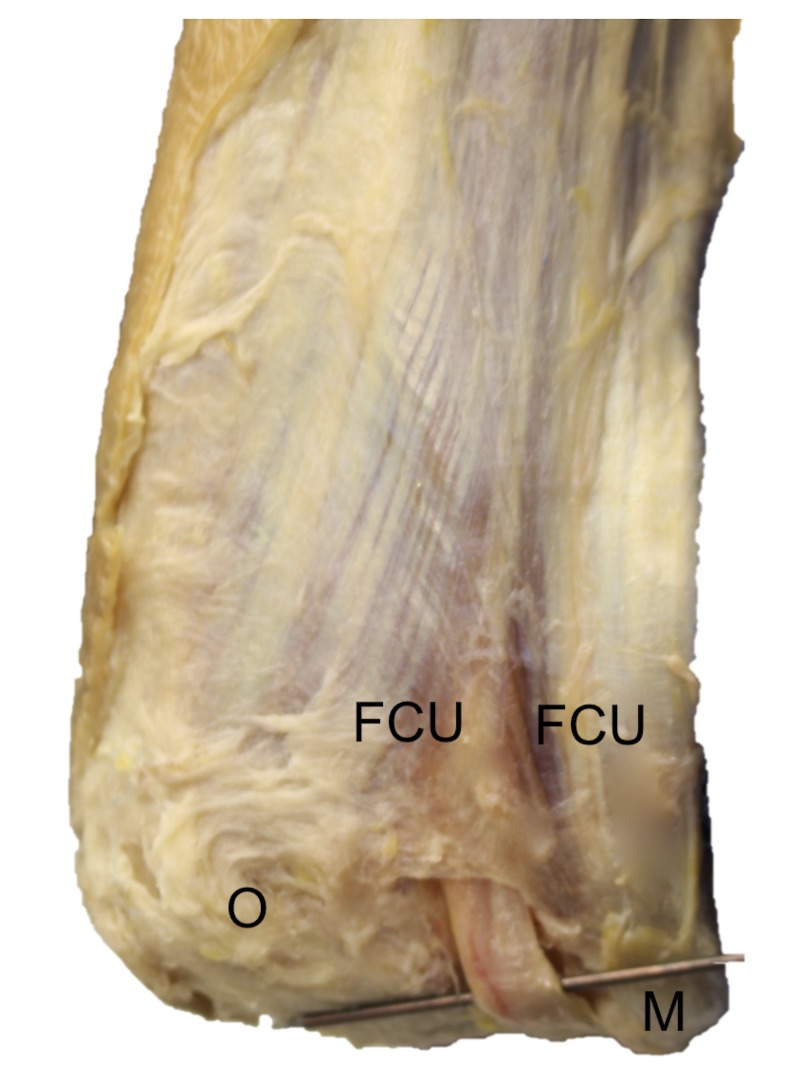
Cadaveric dissection of the right posterior elbow Note the ulnar nerve (crossing pin) as it travels deep into the ligament of Osborne seen here as a triangular connective tissue joining the proximal ulnar and humeral heads of the flexor carpi ulnaris (FCU). For reference, note the medial epicondyle (M) and olecranon (O).

**Figure 2:**
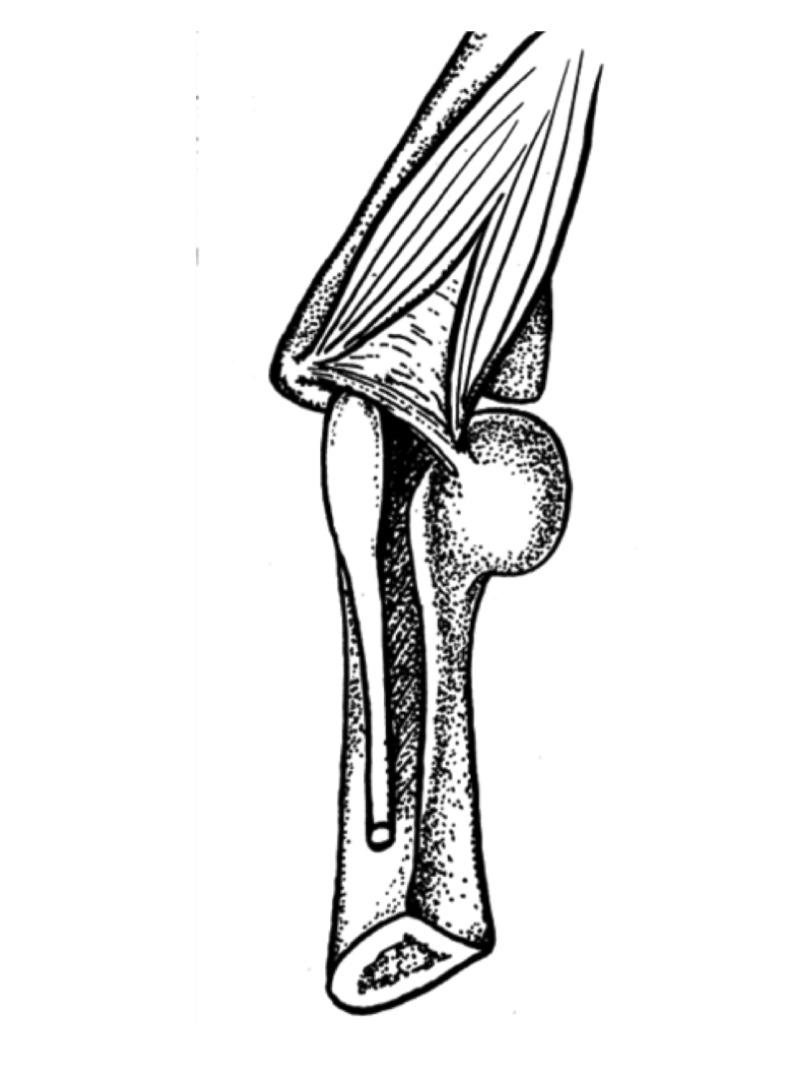
Drawing of Osborne’s ligament Note the entrapment site at the postcondylar groove with a pseudoneuroma of the ulnar nerve proximal to the ligament (Published with permission from [5]).

A comprehensive knowledge of the anatomy of the elbow is essential for diagnosing and treating nerve pathology in this location. With regard to ulnar nerve compression at the elbow, although the exact site is controversial, the cubital tunnel has been implicated as one site of ulnar nerve entrapment [1-6]. In 1957, Osborne described a band of fibrous tissue that spanned between the humeral and ulnar heads of the flexor carpi ulnaris (FCU) muscle and thus formed the roof of the cubital tunnel (Figures 1-2). The cubital tunnel is the fibromuscular canal where the ulnar nerve traverses between the two heads of the FCU with a floor composed of the medial collateral ligament, olecranon, and joint capsule. The so-called Osborne’s ligament has also been referred to as the arcuate ligament of Osborne [7], the cubital tunnel retinaculum [8], Osborne’s fascia [3], Osborne’s band [9], or simply the arcuate ligament or tendinous arch [10] and with Osborne’s publications in the 1950s, was considered as one of the causes of ulnar neuritis [5]. Prior to Osborne’s description of this band of tissue, it was rarely mentioned in the English and French literature [11]. As background, although traumatic ulnar nerve dysfunction was described by Panas as early as 1878, it was not until the early 1900s when Hunt reported spontaneous nerve dysfunction [12-13]. Structural reasons (e.g., ganglion cyst) for these latter presentations were reported by Seddon in 1952 [14]. Five years later, Osborne reported 25 cases of ulnar neuropathy at the elbow and in “almost every case” found compression of the nerve by a fibrous band bridging the two heads of the FCU muscle [15]. Therefore, based on the publications of those such as Seddon and Osborne, non-traumatic structural lesions as a cause of ulnar nerve compression became a widely accepted cause of ulnar neuropathy.

As there are no reviews of this eponymous ligament and its history in the extant medical literature, our aim is to provide such a review and to investigate the anatomy and clinical significance of the ligament.

 

## Review

### History

**Figure 3:**
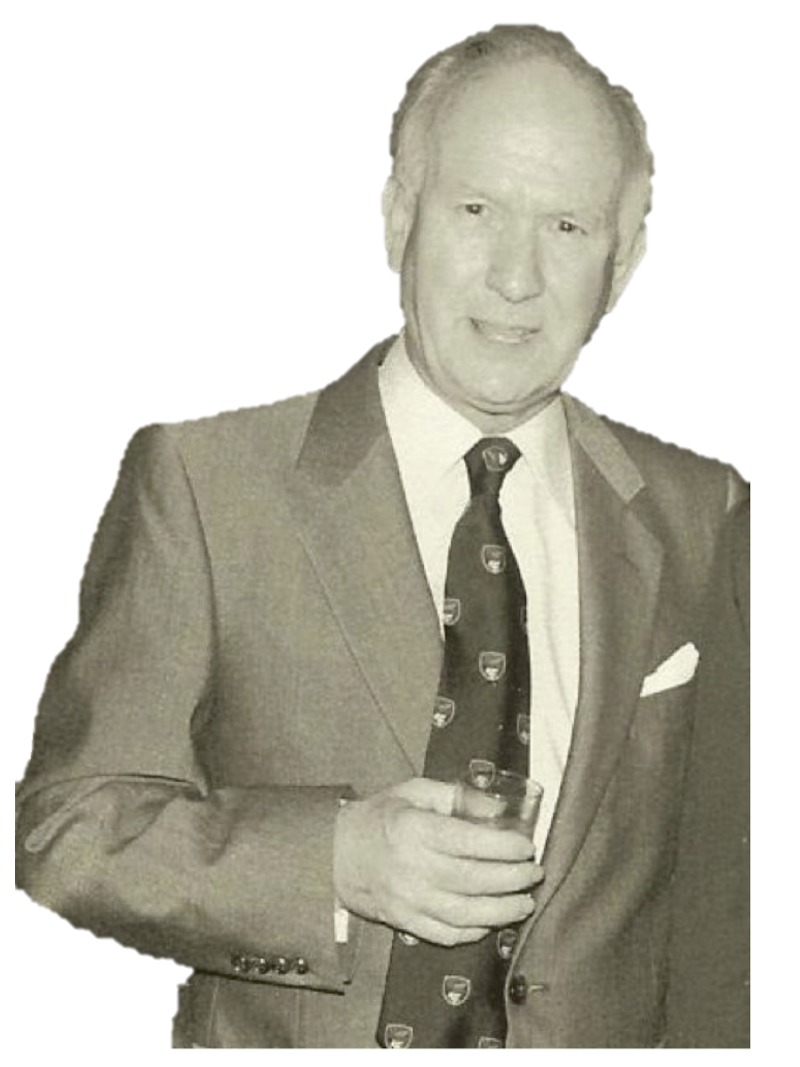
Photograph of Geoffrey Vaughan Osborne (1918-2005) The photograph was taken in the late 1970s (Courtesy: Carrie Osborne).

Geoffrey Vaughan Osborne, MB, ChB, MChOrth, PhD, FRCS, FRCS Ed was born on April 20, 1918, in North Wales (Figure 3). In 1934, at the age of 16, he began his medical training at the Liverpool Medical School. He graduated in 1940 with a distinction in surgery. Although he initially had interest in radiology, his top scores in surgery and the need for more surgeons during the War convinced him to pursue a career as a surgeon (C. Osborne, personal communication, October 2010). Due to his Crohn’s disease, which afflicted him from the second year of his medical training and led to multiple operations over his lifetime, he did not serve in the military. However, he was placed in charge of First Aid in the Southern docks through the worst of the Liverpool Blitz. For the remainder of the War, he practiced surgery and orthopedics in Liverpool. In 1946, he obtained his Fellowship of the Royal College of Surgeons of Edinburgh. The following year, he married and attained his MCh Orth in Liverpool. From 1952 to 1984, he worked as consultant orthopedic surgeon at the Liverpool Royal and Southport Infirmaries. In 1991, he joined the Liverpool Medical Institute and became a life member.

Most of Osborne’s work from the late 1940s to the early 1980s was done in Liverpool (in the University of Liverpool and in Southport), where he was a lecturer in orthopedic surgery [16-17]. Most of his published work centered on orthopedic pathology in the vicinity of the elbow [4-5,18]. He studied osteoarthritis at the hip joint and developed the so-called Osborne-McFarland approach to this region, which was based on the findings that the vastus lateralis and gluteus medius muscles were functionally congruent via the greater trochanter’s periosteum [19-20]. Osborne also carried out several procedures in which the trapezium was removed from patients with carpometacarpal arthritis with good results [21]. He devised methods for decompression of the ulnar nerve at the elbow, repair of recurrent dislocations of the elbow, and the Liverpool (lateral) approach to the hip joint [22]. He was also interested in applying mechanical engineering to orthopedics, invented a variety of instruments and implants for the hip joint, and worked closely with the company Smith and Nephew on an elastic adhesive bandage (Elastoplast) for fixing the Thomas splint, which is used to stabilize a fractured lower extremity. Osborne also worked with Lattimers, an engineering company in Southport, particularly on the development of the Osborne-Ball osteotomy plate, which was primarily used for fixing trochanteric and subtrochanteric fractures.

After half of a century as a prominent and talented orthopedic surgeon he retired from the National Health Service in 1983, but continued doing locums, medical reports, and tribunals until he was 75 years old. He studied computers, mechanics, and machine design and earned his PhD at the age of 78 from Liverpool John Moores University. Upon receipt of his PhD, Osborne said, “At last I am a real doctor” [23]. His field of study for his PhD was machines and machine design; the title of his dissertation was “The History of the Design of the Jobbing Platen Printing Press.” He died in his sleep on April 12, 2005, at the age of 86, just shy of his 87th birthday. 

### Anatomy of Osborne’s ligament

Uniting the triangular muscular interval between the humeral and ulnar heads of the FCU, Osborne’s ligament forms the roof of the cubital tunnel. Mahan, et al., have described this ligament as formed by the fusion of the deep fascia of the FCU and the antebrachial fascia [24]. These authors also mention that the ulnar nerve remains deep to the ligament until it reaches the deep and radial margin of the FCU. Osborne’s ligament has been thought by some to be the remainder of the anconeus epitrochlearis, which is a variant muscle of the elbow [8]. Others have considered it an evolutionarily enhanced version of the anconeus epitrochlearis [25]. Some authors have also stated that when this muscle is present it replaces Osborne’s ligament [26]. Osborne found that it began to become taut at 135^o^ and at 90^o^ of flexion it became very taut and well defined [5,15]. The ligament is about 2.2 cm long from the medial epicondyle to the olecranon and its width is about 4 mm [8,27]. James, et al. measured the thickness of the ligament in eight of their 11 cadaver specimens and found that the mean thickness was 0.15 mm with a standard deviation of 0.08 mm [2]. Macchi, et al. measured a mean thickness of 0.178 mm [7], and on a  magnetic resonance imaging (MRI)-based study, Husarik, et al. found Osborne’s ligament to be thickened in eight percent (five of 60) of subjects [10].

O’Driscoll, et al. referred to Osborne’s ligament as the cubital tunnel retinaculum. It was present in 85% (23 of 27) of their specimens and they categorized it into four types on the basis of morphology and function. In type 0, the ligament was absent; in type Ia, it was lax in elbow extension and taut in full elbow flexion; in type Ib it was tight in positions that did not reach full flexion (90^o^–120^o^); and in type II it was absent with only the anconeus epitrochlearis muscle present. Of their 27 cadaveric elbows, four percent were type 0, 63% were type Ia, 22% were type Ib, and 11% were type II [8]. Macchi, et al. examined the roof of the cubital tunnel and found that it was formed by a tri-laminar structure composed of superimposed layers corresponding to fascia, tendon, and muscle [7]. This multilayered tissue was hyperechoic on ultrasound and had a mean thickness of just less than 1 mm.

The presence of Osborne’s ligament is highly variable. Dellon found it was present in 77% (49 of 64) of his specimens, while James, et al., found it in 91% (10 out of 11) of their cadavers [2,25]. However, other studies have observed it in as few as eight percent (one of 12) or as many as 100% (39 of 39) of the specimens [27-28].

### Surgical/clinical significance

The ulnar nerve is the nerve most commonly involved in entrapment syndromes at the elbow. Osborne’s ligament has frequently been implicated in the etiology of ulnar neuropathy [4-6,9,16,29]. It has been proven that the cubital tunnel’s volume deep to the ligament decreases as the elbow flexes [2]. For this reason, Osborne’s ligament can be involved in the development of some cases of ulnar nerve compression [5,16,18]. Pathologically, it has also been implicated in some disease processes like increased laxity of Osborne’s ligament in patients with Ehlers-Danlos syndrome, which can lead to entrapment and ulnar neuropathy [30].

To transect the band that was eventually named after him, Osborne decompressed the ulnar nerve using a small three-inch incision over the elbow and parallel to the ulnar nerve. The proximally swollen ulnar nerve was then identified and mobilized, the subcutaneous fat in the region was sutured over the nerve, and the skin was closed [5]. He found that several cases of idiopathic ulnar neuropathy, which accounted for more than 10% of all his cases of ulnar neuropathy, were relieved after the ligament was surgically divided. 

Osborne’s ligament can be seen on both ultrasound and MRI [3,29,31-32]. Although controversial and not accepted uniformly, the scratch collapse test is used to gauge its tension [33]. For this examination, the patient sits with a flexed elbow at 90^o^ and fingers pointing toward the examiner who attempts to rotate the forearm medially and takes note of the patient’s baseline “resistance.” The area over the proposed site of entrapment is then stroked and the test is repeated. An observable decrease in “resistance” indicates a positive test, while simultaneously identifying the site of impingement in the vicinity of Osborne’s ligament [9]. Cheng, et al. used the scratch collapse test for diagnosing cubital tunnel syndrome with 89% accuracy [34]. Using this test, Davidge, et al. found that the primary entrapment point of the ulnar nerve was Osborne’s ligament in 80% of the patients examined in their prospective study [33]. Lastly, one proposed etiology of the so-called snapping triceps syndrome, i.e., dislocation of the ulnar nerve with elbow flexion, is a congenital absence of Osborne’s ligament [16,35].

## Conclusions

To our knowledge, no past publication has reviewed the history of Osborne’s ligament and very few authors have studied its anatomy in any detail. This tissue is apparently involved in many cases of ulnar nerve compression at the elbow. Therefore, a good understanding of its anatomy is important for those who diagnose or operate on lesions of the ulnar nerve in this region. The early contributions of Osborne to our understanding of ulnar nerve compression cannot be overestimated.
